# Evaluation of the effectiveness of a semi-finished occlusal appliance – a randomized, controlled clinical trial

**DOI:** 10.1186/1746-160X-9-5

**Published:** 2013-01-25

**Authors:** Tobias Ficnar, Claudius Middelberg, Bernd Rademacher, Stefan Hessling, Raphael Koch, Ludger Figgener

**Affiliations:** 1 Department of Prosthetic Dentistry and Biomaterials, University Hospital Münster, Alber-Schweitzer-Campus 1, Building W 30, 48149, Münster, Germany; 2 Department of Orthodontics, University Hospital Münster, Alber-Schweitzer-Campus 1, Building W 30, 48149, Münster, Germany; 3 Institute of Biostatistics and Clinical Research, University of Münster, Albert-Schweitzer-Campus 1, Building A11, 48149, Münster, Germany

**Keywords:** Myofascial pain, Occlusal appliances, Pain, Randomized controlled trial, Temporomandibular disorders

## Abstract

**Introduction:**

Painful temporomandibular disorders (TMDs) are usually treated with physiotherapy, self-exercises, medication-based therapy and splint therapy. For splint therapy different types of splints are available. Therefore this randomized controlled study compared the effectiveness of a semi-finished occlusal appliance (SB) with a laboratory-made occlusal appliance (SS) in myofascial pain patients.

**Method:**

The trial subjects allocated to the experimental groups with the (SB) occlusal appliance and those provided with a laboratory-made occlusal appliance (SS) did, in addition, receive conservative treatment (self-exercises, drug-based and manual therapy). The control group was given conservative therapy (CO) only. Overall, a total of 63 patients participated in the study with each group consisting of 21 subjects.

**Results:**

When the first follow-up examination took place (14 days after splint insertion) mouth opening within the SB group was significantly enlarged. When the second examination was conducted (2.5 months after splint insertion) mouth opening was significantly enlarged in both splint groups when compared with the initial value. In the control group, no significant enlargement of mouth opening was detected. At no point there was a significant reduction in the number of pressure-sensitive areas of the TMJ. On palpation of the masticatory muscles however, a significant reduction in the number of pressure-sensitive areas could be observed within the CO group and the SS group after 2.5 months.

When comparing pain reduction (muscle/joint pain) and mouth opening, no significant differences could be detected between the treatments.

**Conclusion:**

The results suggest that TMD should be treated conservatively. In cases of restricted mouth opening, the additional use of occlusal appliances can eliminate the patient’s discomfort more quickly. In this context, the tested, semi-finished occlusal appliance appears to offer an immediately available, temporary alternative to laboratory-made splints.

## Introduction

*Temporomandibular disorder* (TMD) represents a problem in terms of health economics. Currently available data suggests that, on average, 9.7% of the population suffers from conditions covered by RDC/TMD group I diagnosis (myofascial pain) and 11.4% from conditions covered by RDC/TMD group IIa diagnosis (disk displacement with reduction) [1] with the risk of women developing these disorders being approximately 1.5 to 2 times higher than that of men. 80% of patients treated for TMD are women [2-4].

Dysfunctions and functional disorders of the craniomandibular system are characterized by a multitude of symptoms that are similar to complaints that typically affect the human locomotor system. Examples are discoordination of synergistic and antagonistic muscle groups, various myogenic types of pain (e.g. myalgia), muscular hypertrophies and/or hypotrophies as well as arthrogenic conditions with dysfunctional physiological joint positions and, finally, secondary pathological changes of the temporomandibular joints. In addition, traumatic, psychological and orthopedic cofactors are being discussed [5].

During the search for an etiological explanation of TMD, the initial assumption was that the cause of TMD-associated complaints was primarily a dysfunctional occlusal relationship between the upper and lower jaw and a resulting change in the position of the lower jaw – or the condylus in the articular fossa [6]. Meta-analyses however were able to show that this purely biomedical approach did not do justice to TMD [7]. Nowadays there is no doubt that biomedical as well as psychological and psycho-social impact factors are involved in the development of TMD [8]. This etiological model of explanation, which has by now been accepted, was first published as early as 1992 by Dworkin and LeResche. Their “research diagnostic criteria for temporomandibular disorders” (RDC/TMD) are clearly the gold standard of TMD diagnostics in the current scientific debate.

For a long time the assumption was that the adjusted surface of the occlusal splint was essential for its effectiveness as it led to the reconstitution of an “ideal” and/or “physiological” position of the lower jaw [9,10]. Over the past few years however, a multimodal treatment concept has come to the fore that consists of elements such as temporary reversible changes of occlusion with the help of occlusal splints but also of manual therapy, biofeedback, self-exercises and pharmacotherapy. Furthermore, psychotherapeutic and cognitive behavioral treatment procedures are being used [11]. With regard to splint therapy, the more recent evidence confirms an effectiveness of ready-made occlusal appliances or interceptors without adjusted surfaces for the treatment of TMD-associated complaints [12,13].

The effectiveness of the mentioned occlusal appliances is currently explained with the help of the integrated neurobiological model [14] whose basic idea lies in the heterogeneous activation of the masticatory muscles [15]. This is inspired by the thought that a random change in jaw relations leads to an altered activation pattern of the masticatory muscles that relieves previously overstrained areas.

**Figure 1:**
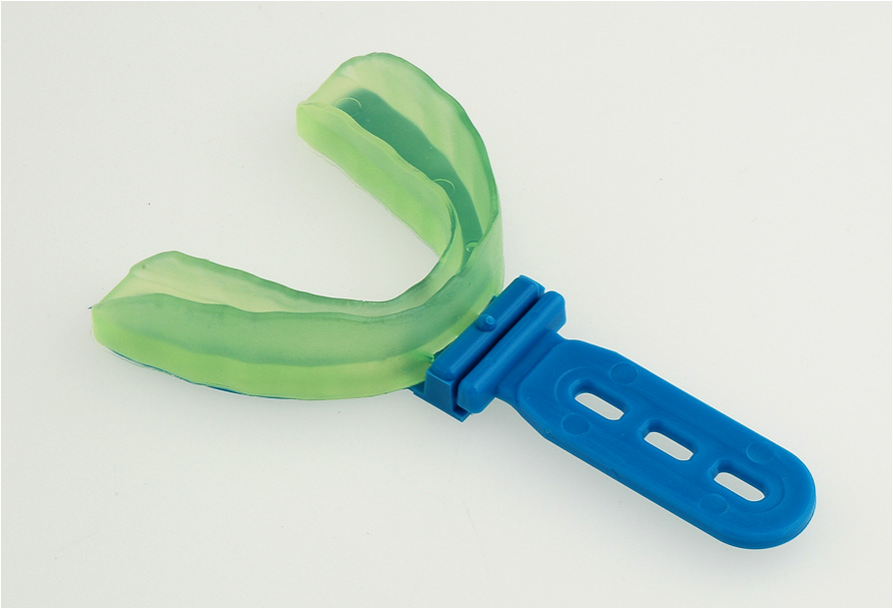
**Semi-finished occlusal appliance prior to adjustment.** The blue occlusal liner and the handle are removed after adaptation.

**Figure 2:**
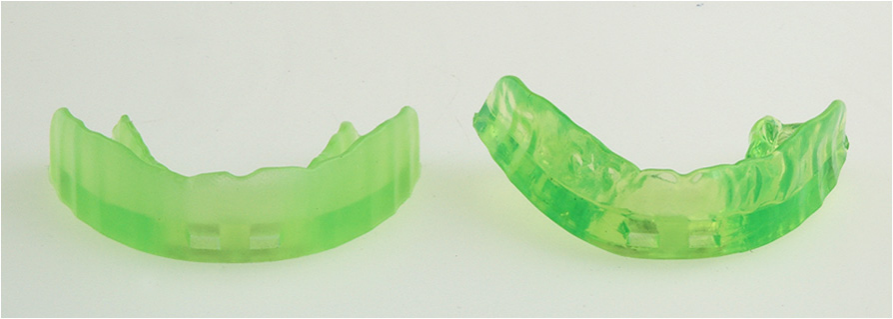
**This shows the difference between an occlusal appliance that has not yet been individualized (left) and one that has been individually adjusted.** The adapted occlusal appliance has changed shape in line with the compensation curves.

**Figure 3:**
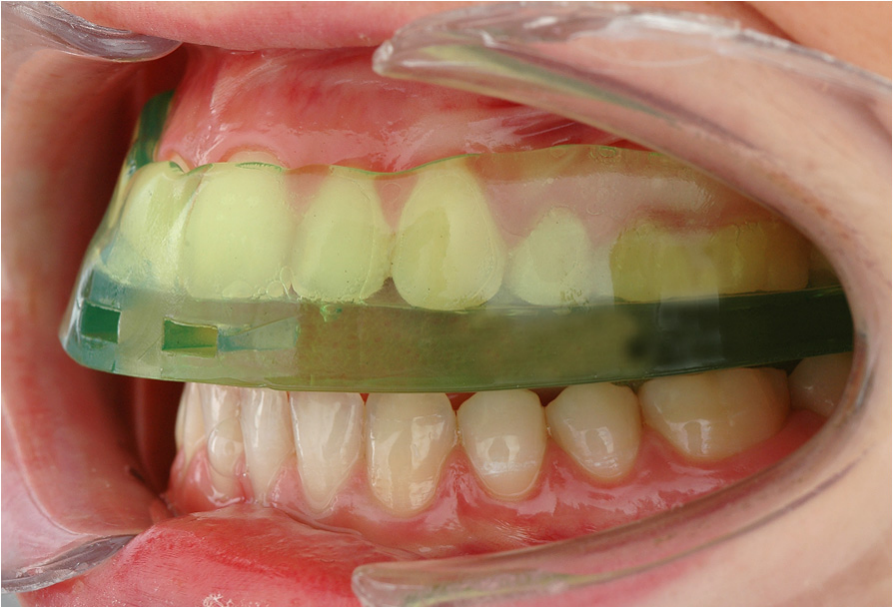
**Adapted, semi-finished occlusal appliance that has been placed onto the maxillary teeth.** All mandibular teeth occlude against the planar underside of this splint.

The semi-finished occlusal appliance called “SOLUBrux®”(W3 Solutions, 4ch.delaBobinette, CH-1263 Crassier, Switzerland) (Figure 1), whose effectiveness has been tested in this study, is made of malleable thermoplastic material on an ethyl vinyl acetate basis, which, according to the manufacturer’s instructions, it meant to be fitted to the upper jaw after being heated in water. The occlusal appliance is designed in such a way that the sections that face the buccal and oral tooth surfaces are thinner than those facing the occlusal surfaces. When submerged in water, the thin sections soften to a greater extent so that they adjust to the shape of the teeth whilst the thicker sections change their shape only slightly. During the fitting process, a so-called “occlusal liner” is clipped onto the part of the occlusal appliance that faces the lower jaw. This ensures that the splint adapts to the compensation curves while, at the same time, preventing an occlusal adjustment that would leave cusp imprints (Figures 2, 3).

### Trial subjects, material and method used

The trial subjects were recruited in 2009 and 2010 in the Department of Prosthetic Dentistry and Biomaterials and the Department of Orthodontics of the Center for Dental, Oral and Maxillofacial Diseases of Münster University Hospital.

The screening was performed on female and male patients aged 18 to 80 years who had been referred there due to TMD-associated complaints.

The patients who were deemed potentially suitable for participation in the study were, in advance, told of the study design during an introductory session with an investigator in charge of monitoring the study, and received an information leaflet. The trial subjects agreed to take part in the study by signing a consent form.

The research project was approved by the ethics commission of the Regional Medical Association (Ärztekammer) of Westfalen-Lippe and the Medical Faculty of the Westphalian Wilhelms University of Münster.

Case number estimates carried out by the Institute for Biometrics and Clinical Research of Münster University Hospital established that a minimum number of 15 trial subjects was required per group.

### Composition of the group of trial subjects

Inclusion criteria: Included were patients who had been diagnosed with RDC/TMD Ia or Ib (myofascial pain) also in combination with arthralgia (IIIa) and/or disk displacement with reduction (IIa) and a maximum “von Korff” pain grade of I (functional pain with low levels of intensity) to II (functional pain with high levels of intensity).

Exclusion criteria: Excluded were patients with other RDC diagnoses, polyarthritides, full denture users, patients with severe general diseases, with radiation of the maxillo-facial area, patients who had suffered maxillo-facial trauma, those with allergies against plastic dentistry materials, with mouth openings ≤ 20 mm and those suspected of neoplasias and mental health problems.

Patients, however, who had unsuccessfully undergone splint therapy or other CMD treatments in the past were not excluded.

Termination criteria: Patients suffering from an intolerance to the splint material and whose mouth opening was increasingly restricted (≤ 20 mm) stopped taking part in the study.

**Figure 4:**
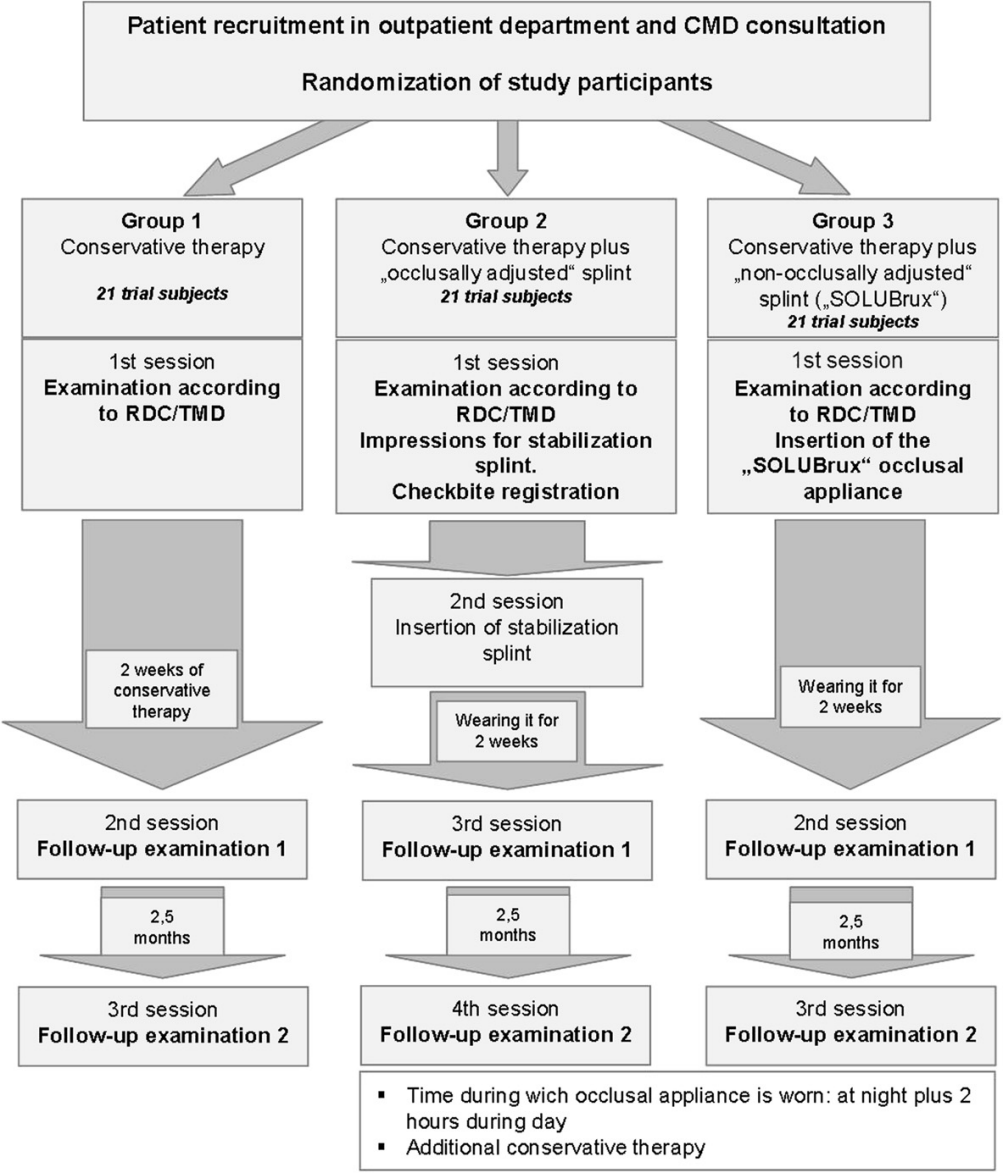
Diagram of study development.

The overall group of trial subjects consisted of 63 persons. They were assigned to the three different sub-groups via randomization (21 persons per study group) (Figure 4):

### Group 1

Conservative therapy: Included the use of self-exercises (muscle exercise form according to Prof. Schulte, self-massage techniques, mouth opening exercises), medication-based therapy using non-steroidal anti-inflammatory drugs (NSAID), muscle relaxants as well as manual therapy.

### Group 2

Conservative therapy. In addition, a laboratory-made stabilization splint was produced. As part of this splint therapy, a SAM® face-bow was fitted and, in order to establish the centric occlusion position, a bite registration was taken using Beauty Pink® wax as registration plate and Aluwax®.

### Group 3

Conservative therapy. In addition, the SOLUBrux® splint was fitted and inserted. This prefabricated, semi-finished occlusal appliance made from malleable thermoplastic material was produced and/or fitted in line with the manufacturer’s instructions.

To this end, the combined occlusal liner/fork handle was fully inserted into the appropriate opening on the front of the SOLUBrux® splint. This was followed by an examination of its fit in the mouth. The SOLUBrux® splint could be reduced in size with a scalpel or a pair of scissors. To ensure later adaptation, the patient was told to practice pressing his tongue against the anterior palate and, by doing so, creating a vacuum in the oral cavity.

Subsequently, the SOLUBrux® splint was heated in boiling water for 12–15 seconds.

After taking it out, the hot water was removed from the SOLUBrux® splint, which was then adjusted to the mouth of the patient as described.

Patients in group 2 and 3 were told to wear the occlusal appliance every night and two hours during the day.

### Treatment protocol

The patients were examined using a standardized protocol (RDC/TMD). This includes establishing active and passive incisal edge distance, the extent of excursive movements, the deviation of jaw opening from the mid-line, palpation of the masticatory muscles and temporomandibular joints from lateral and dorsal as well as auscultation of temporomandibular joint noise.

As the occlusion is not recorded in the RDC/TMD, it was in addition documented with the help of the occlusion protocol taken from functional status of the German Society for Functional Diagnosis and Therapy (DGFDT). This records tooth contact patterns in habitual, centric static as well as dynamic occlusion.

### Follow-up and collection of data

Findings were collected and documented at the start of each patient’s therapy, two weeks after therapy commencement as well as three months after the start of the therapy. In case of study group 2, a further day was added for the insertion of the laboratory-made occlusally adjusted splint. In this group too, the second time that findings were collected and documented was two weeks after the insertion of the occlusally adjusted stabilization splint.

### Principles of statistic evaluation

#### Target parameters

The primary aim of the study was to compare the three treatment groups with regard to pain reduction in the palpation-sensitive masticatory muscles. The primary outcome parameter was the difference in the number of affected areas between the start of the study and the first follow-up examination.

Apart from the primary evaluation, additional exploratory evaluations were carried out. These included changes concerning the reduction of pain between the commencement of the study and the second follow-up examination as well as changes within each treatment group. In addition, further sections of the RDC/TMD questionnaire were evaluated. The resulting p-values and confidence intervals were interpreted as descriptive measurements. Inferential statistics are intended to be exploratory (hypotheses generating), not confirmatory, and are interpreted accordingly. I.e., p-values are interpreted in Fisher’s sense, representing the metric weight of evidence against the respective null hypothesis of no effect. These findings will be used to generate new hypotheses.

#### Statistical methods

The statistical evaluation of the primary target parameters was performed as a confirmatory analysis that involved the use of non-parametric statistical procedures. First, Kruskal-Wallis tests were performed to establish any global differences between the three treatment groups. The global significance level was set to α = 5%. In the case of significant test results, post-hoc analyses using two-sided Mann–Whitney U tests were conducted. Due to multiple comparions p-values were adjusted using Bonferroni correction.

Differences in the extent of vertical movement, extraoral muscle palpation and palpation of the joints within each treatment group between the examinations were analyzed using the Wilcoxon test with Bonferroni correction.

All statistical analyses were performed by the Institute of Biostatstics and Clinical Research University of Münster using the statistical software packages IBM SPSS® Statistics 20 for Windows (IBM Corporation, Somers, NY, USA) and SAS Statistical Software (SAS v. 9.2; Statistical Analysis System SAS Institute, Inc., Cary, NC, USA.

## Results

### Demographic results

**Figure 5:**
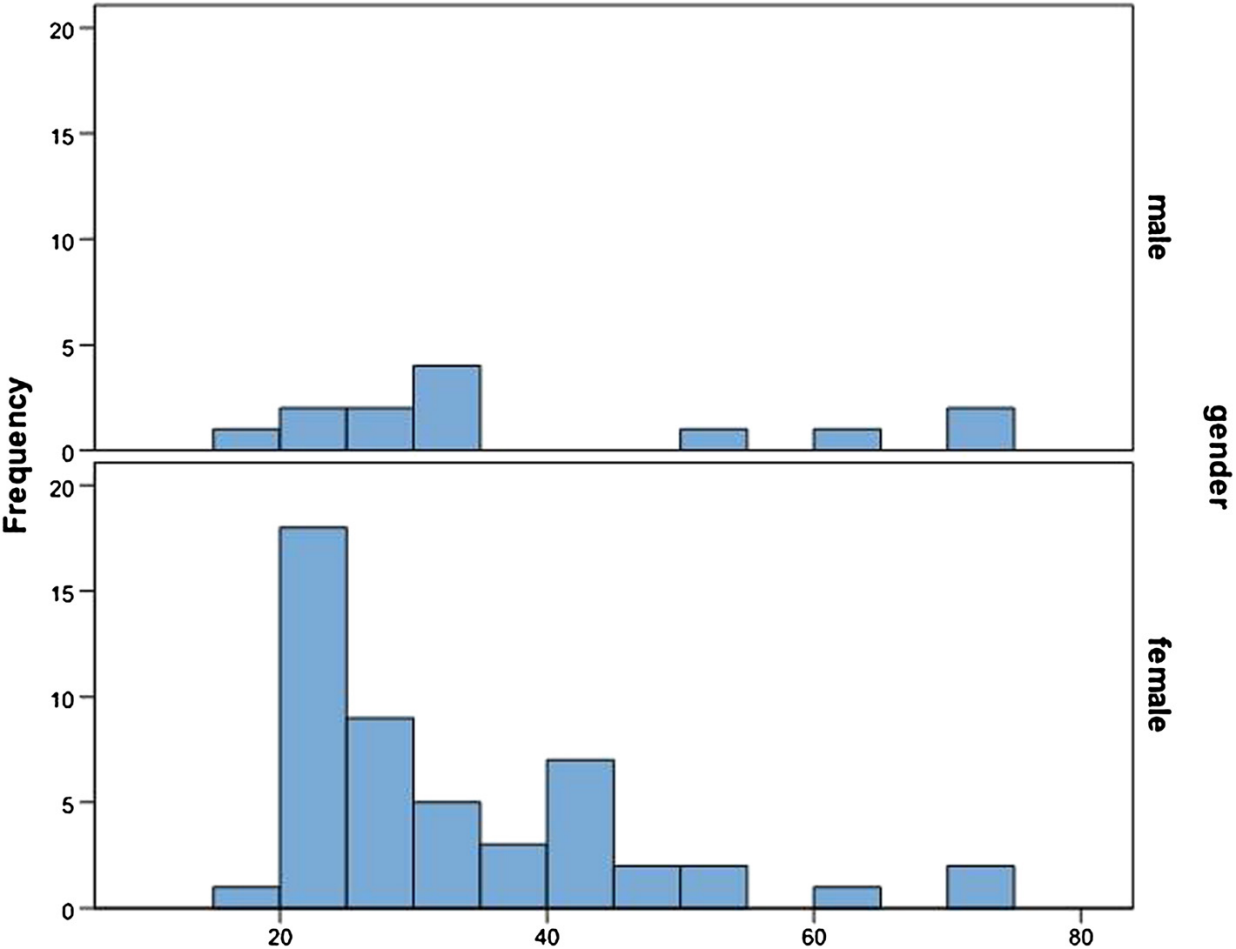
Age-dependent gender distribution.

The overall gender distribution was 79.4 percent in female and 20.6 percent in male trial subjects. The median age was 34.66 years. The average age of the female patients was 33.38 years and that of the male participants was 39.60 years (Figure 5).

### Dropout

All trial subjects of the SB group finished the study whilst in the CO group two participants and in the SS group three participants dropped out (Table 1). One of the drop-outs was due to an adenoid cystic carcinoma spreading in the aural/temporomandibular joint area, which was detected during the course of the study, whereby one drop-out criterion was met. Persisting complaints and increased swelling above the temporomandibular joint made the investigator carry out further diagnostic imaging procedures.

**Table 1:** Processed cases

	**Cases**		
	**Valid**		**Missing**
**Therapy**	N	Percent	N
conservative (CO)	19	90.5%	2
occlusally adjusted (SS)	18	85.7%	3
not occlusally adjusted (SB)	21	100%	0

### Examination results

During none of the follow-up examination days statistically significant differences could be established between the three groups concerning the examined parameters. Generally, however, it was possible to observe a reduction in the pressure-sensitive areas upon palpation of the muscle and temporomandibular joint area as well as an increase in the extent of vertical movement in all three groups.

However, when comparing these parameters within the groups at the different follow-up examination times, some statistically significant differences could be established.

**Figure 6:**
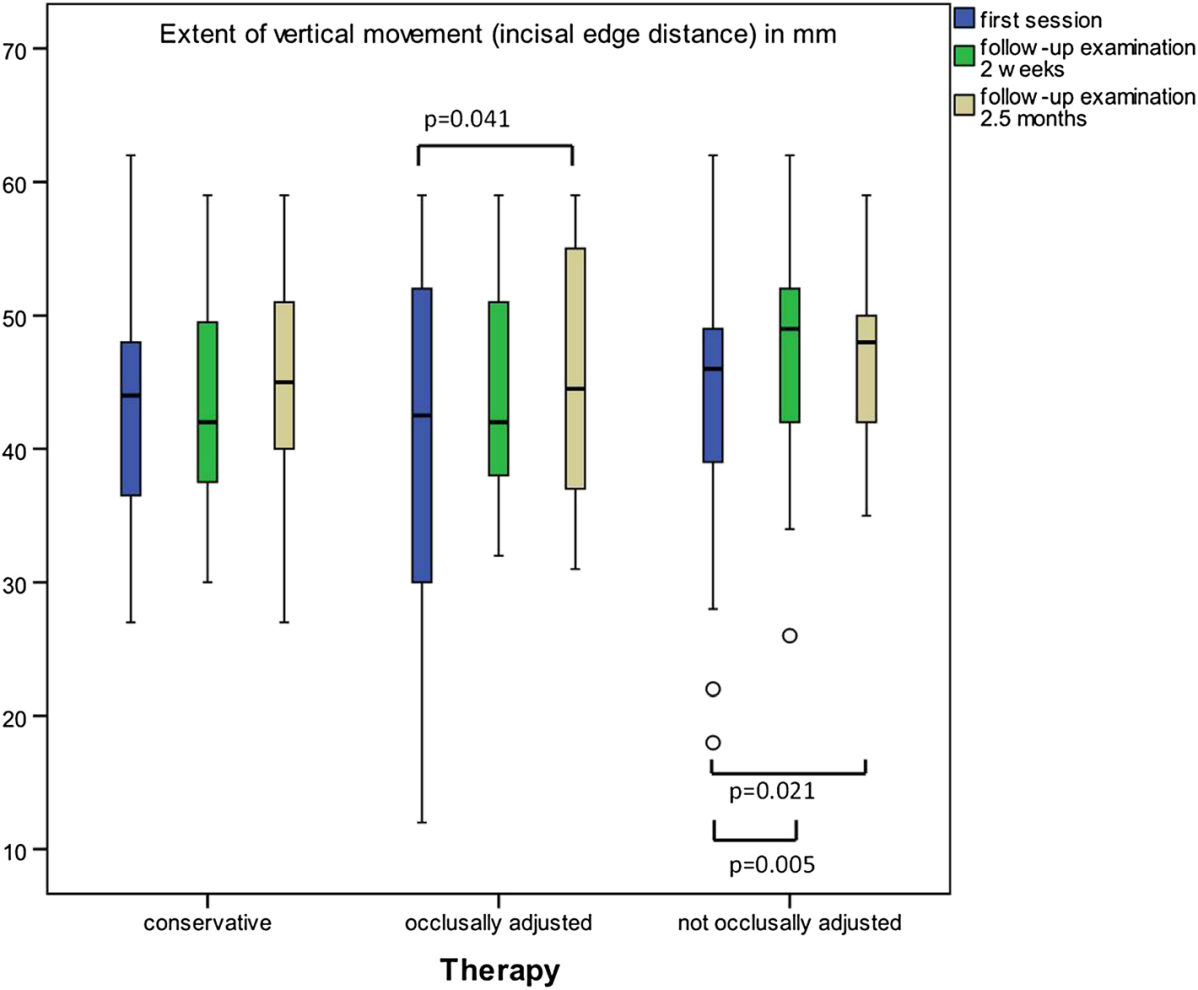
**Extent of vertical movement.** Significant differences after a Bonferroni correction of the Wilcoxon test within the groups. (p < 0.05; brackets mark significant differences).

Concerning the extent of pain-free, active vertical movement of the lower jaw, no statistically relevant difference was found within the CO group whilst in the SS group there was a difference between the initial findings and the final examination after 2.5 months (P = 0.041), and in the SB group there was a difference both between the initial findings and the two-weekly follow-up examination (p = 0.004) and between the initial findings and the final examination (p = 0.021) (Figure 6).

In the SB group the increase in active mouth opening accompanied by pain between the initial findings and the two-weekly follow-up examination was significantly enlarged (p = 0.025).

**Figure 7:**
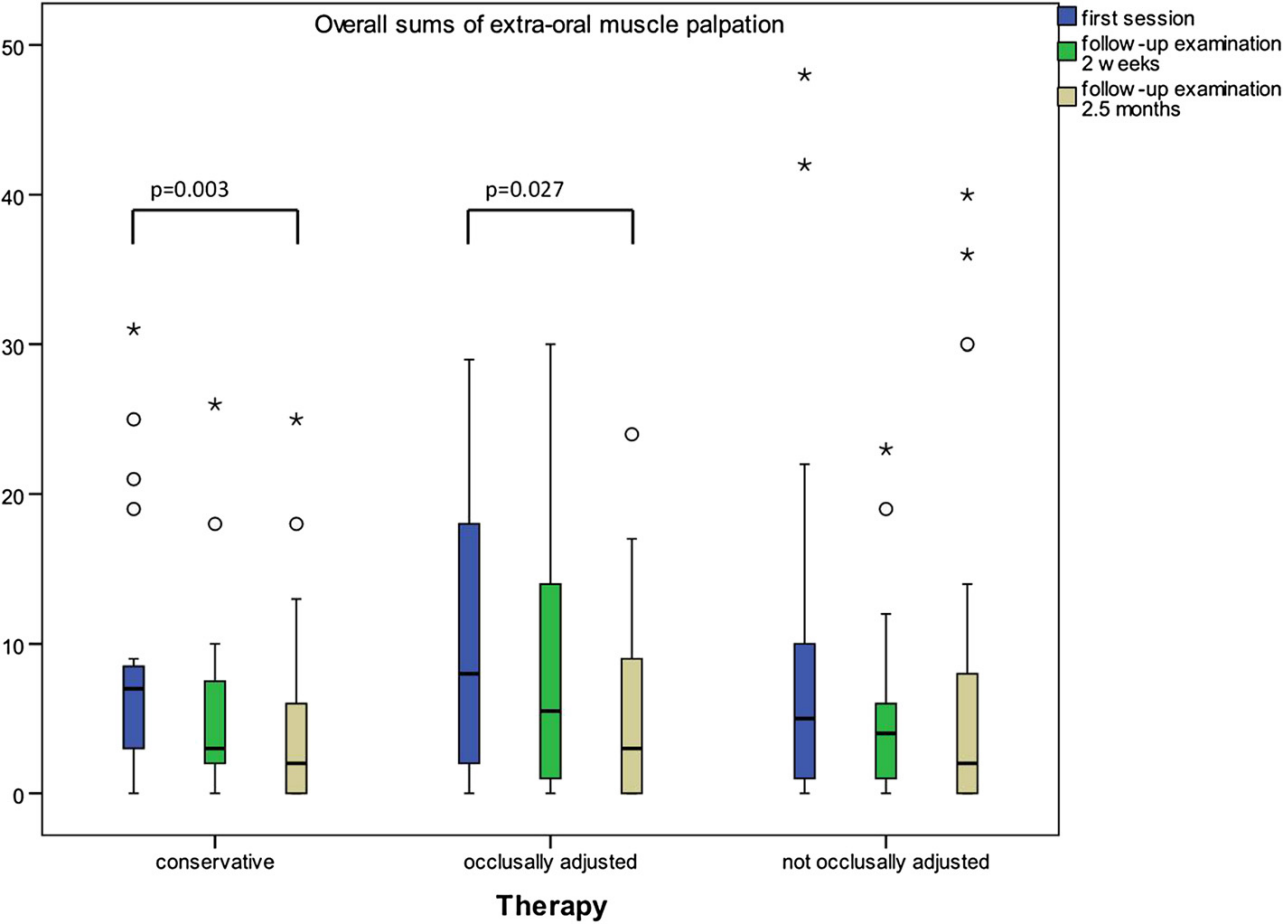
**Reduction in the number of pressure-sensitive areas upon muscle palpation.** Significant differences after a Bonferroni correction of the Wilcoxon test. (p < 0.05; brackets mark significant differences).

As to the overall number of extraoral muscle palpation areas, an overall reduction in the pressure-sensitive areas could be observed in all three groups whilst significant changes between initial and final findings only occurred within the CO (p = 0.002) and the SS (p = 0.027) groups (Figure 7).

**Figure 8:**
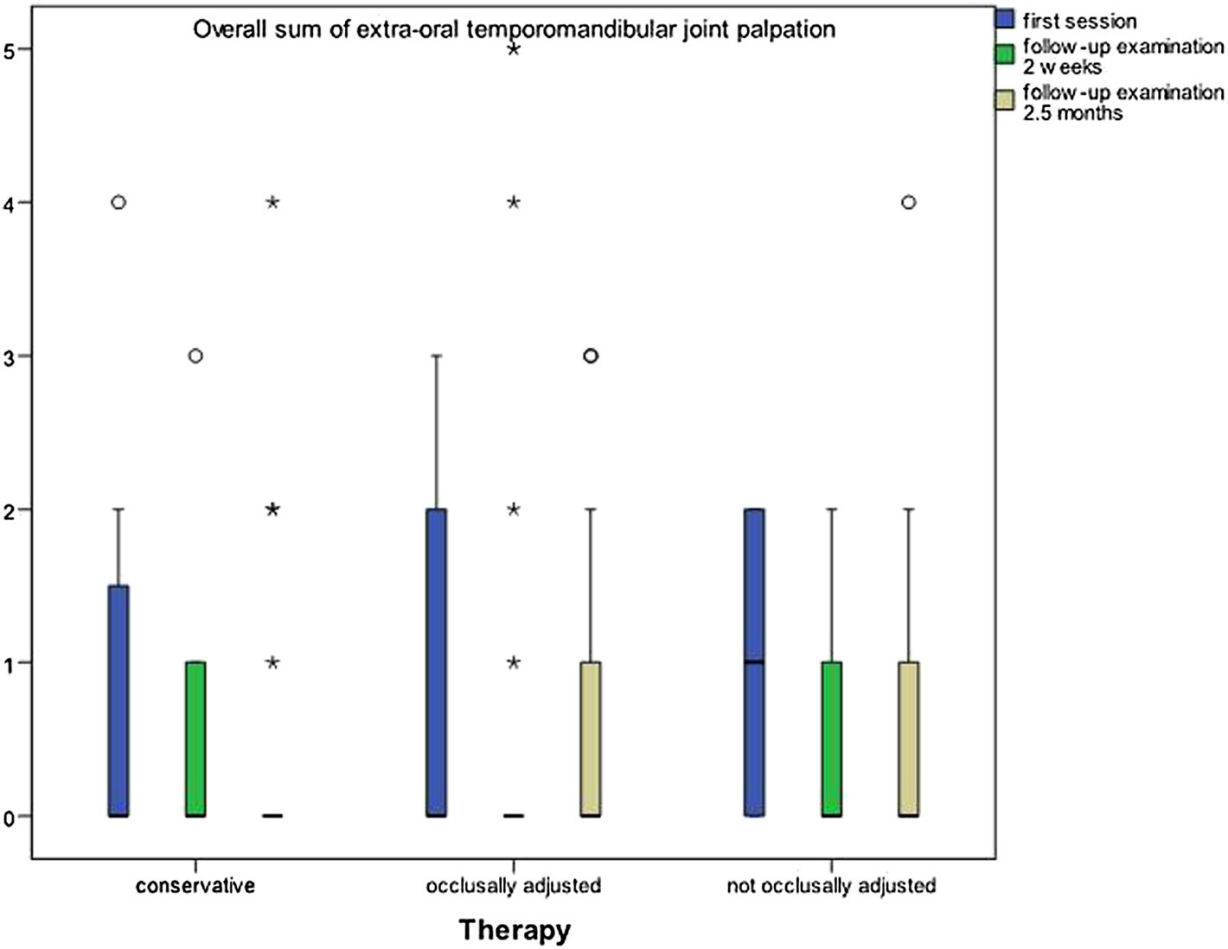
**Reduction of pressure-sensitive areas during palpation of temporomandibular joints.** No statistically significant differences within the groups. (p < 0.05; brackets mark significant differences).

Palpation of the temporomandibular joints did not show any statistically significant differences regarding the reduction of pressure-sensitive areas within the three groups. There was a conspicuously large number of blips. However, a non-significant reduction still occurred in all groups (Figure 8).

## Discussion

The gender and age distribution of the study participants correlates with the results of epidemiological studies on TMD. The presumption, for example, is that 80 percent of patients who are treated for TMD are women [2,16]. John [17] established in 2001 that the frequency of women receiving TMD therapy is double that of men. In our study the percentage of women was 79.4% while that of men was 20.6%.

Clinical patient populations primarily consist of middle-aged adults [18]. According to publications, functional disorders typically peak when people are in their thirties or forties with a disproportionately high number of female sufferers. These figures are identical with the median age of our study participants.

Our study did not establish any significant differences between the three groups with regard to therapeutic effectiveness.

These results concur with the studies conducted by Truelove 2006 [12] and Alencar 2008 [19]. Truelove compared the results of conservative therapy with those achieved with a low-cost sport mouth guard added to conservative therapy and those obtained with a laboratory-made “expensive” splint that was added to conservative treatment. As in our study, pain reduction and increased mouth opening was observed in all three groups albeit without significant differences. His conclusion was to treat CMD with low-cost conservative therapy (e.g. self-exercises, drug-based therapy). When comparing results he did not see that treatment based on adjusted occlusal appliances provided any additional benefit.

Using a double-blind controlled clinical study, Alencar [19] compared hard, occlusally adjusted splints with soft ones as well as with splints without antagonistic contacts in patients with myofascial pain of the masticatory muscles. Here too, all three groups noticed a reduction in pain, which led to the conclusion that low-cost therapies such as self-exercises are preferable to more expensive splint-based therapies.

Our results and the above mentioned studies contradict popular and plausibly documented models as well as our own experience according to which occlusal splints have a therapeutic effect on patients suffering from myogenic CMD. Schindler and Türp 2011 [20] state that, based on the highest levels of evidence, a specific therapeutic effect could be confirmed in this subgroup. According to them, particular uncomplicated complaints that are limited to the masticatory muscles respond well to occlusal splint treatment.

Based on a meta-analysis, Fricton concluded in 2010 that hard, occlusally adjusted splints had a better therapeutic effect than placebo splints or no treatment at all [21].

There are several hypotheses concerning the efficacy of occlusion splints such as the introduction of changes affecting behavior and awareness [22] as well as the re-organization of functional intramuscular patterns based on the heterogeneous activation of the masticatory muscles, which reduces the strain on damaged muscle sections [23,24].

Two weeks after splint insertion, an MRI scan detected a neurocerebral effect. It found decreased activity in the somatosensory (S1) and primary cortex (S2) as well as in the insular cortex (higher-level pain perception) and an activation of parietal brain sections that are used for the control of precise coordinated movements [25].

Splints have also proven to be therapeutically effective in cases of disk displacement [26-28]. Compared with untreated control groups, the treatment success of splint therapy was noticeable much earlier.

In view of the above, the question necessarily remains as to why the occlusal splints used in this study did not result in the desired additional therapeutic effect concerning muscle and temporomandibular joint pain.

What would need to be discussed is the choice of a dental hospital with TMD consultation hours as the place of treatment. We must ask ourselves whether the patients who were examined there are a representative section of all patients with TMD symptoms or whether, from the very beginning, the patient selection led to an unavoidable bias effect.

Concerning their prognosis, patients referred by their dentists to a dental hospital or who decide to go there of their own accord (e.g. because they are not satisfied with the current treatment provided by their dentist) could be different from patients who never consulted a doctor regarding their complaints or whose treatment their dentist was confident enough to provide.

It is a well-known fact that patients who are referred to specialist pain clinics complain about more persistent pain and greater functional restrictions are more likely to have been through unsuccessful prior treatments and generally benefit least from any intervention [29]. The results of this study may therefore only be transferred to similar patient groups.

Within the SB and SS groups, significantly increased mouth opening could be observed with this effect occurring even earlier in patients fitted with a SOLUBrux® occlusal appliance.

There could be various explanations for this effect. When splints are fitted, the mouth needs to open wider, which could lead to a stretching effect. This could also explain why the effect is faster when a SOLUBrux® occlusal appliance was used, because it is significantly thicker than a normal stabilization splint. In addition, the splints could have resulted in a relaxation of the tense and shortened masticatory muscles, which led to a normalization of muscle tone and an enlarged mouth opening. Even certain distractive effects of the splints due to an increase in the vertical dimension are possible, which could have reduced the strain on the temporomandibular joint structures. Furthermore, the immediate availability of the splint could have had a positive effect on mouth opening because other tests have shown that an early use of splint therapy causes a greater increase in mouth opening [30].

## Conclusion

This randomized controlled study did not establish significant differences regarding pain reduction (muscular/joint pain) and mouth opening between the various therapeutic approaches.

These results suggest that TMD should initially be treated with conservative therapy consisting of self-exercises as well as drug-based and manual treatment.

It must, however, be said that a significant improvement of mouth opening was observed within the SB and SS groups, which was missing within the CO group. This effect occurred more rapidly with the semi-finished SOLU-Brux® occlusal appliance than with the laboratory-finished occlusion splints. Hence, the use of occlusal appliances, particular in cases of restricted mouth opening, continues to make sense as it alleviates complaints more quickly. The immediate availability of semi-finished occlusal appliances, which can directly be adapted to the individual patient, may be an advantage.

The question is how far results that have been obtained in a specialist facility can be transferred to a general dentist’s practice considering that bias effects must be expected that originate from the patient population itself. This requires further research in order to establish to what extent such differences actually exist.

## Competing interests

The expenses for this study were payed by Jaxeurope (Jaxeurope, Eifelstraße 15, 65232 Taunusstein, Germany). No remunerations have been paid. The authors declare that they have no competing interests.

## Authors’ contribution

TF made substancial contributions to concept and design of the study, performed clinical examination and wrote the main part of the manuscript. CM made also substancial contributions to concept and design of the study and performed clinical examination. BR and SH performed clinical examination and made acquisition of data. RK performed the statistical analysis and interpretation of data. LF suggested the original idea for the paper and has given final approval of the version published. All authors read and approved the final manuscript.
